# Genome-wide DNA methylation dynamics during epigenetic reprogramming in the porcine germline

**DOI:** 10.1186/s13148-021-01003-x

**Published:** 2021-02-03

**Authors:** Isabel Gómez-Redondo, Benjamín Planells, Sebastián Cánovas, Elena Ivanova, Gavin Kelsey, Alfonso Gutiérrez-Adán

**Affiliations:** 1 Department of Animal Reproduction, INIA, Madrid, Spain; 2 Physiology of Reproduction Group, Department of Physiology, Universidad de Murcia, Campus Mare Nostrum, Murcia, Spain; 3 Instituto Murciano de Investigación Biosanitaria, IMIB-Arrixaca-UMU, Murcia, Spain; 4 Epigenetics Programme, The Babraham Institute, Cambridge, UK; 5 Centre for Trophoblast Research, University of Cambridge, Cambridge, UK

**Keywords:** Embryo, Primordial germ cells, Methylation reprogramming, Whole-genome bisulphite sequencing

## Abstract

**Background:**

Prior work in mice has shown that some retrotransposed elements remain substantially methylated during DNA methylation reprogramming of germ cells. In the pig, however, information about this process is scarce. The present study was designed to examine the methylation profiles of porcine germ cells during the time course of epigenetic reprogramming.

**Results:**

Sows were artificially inseminated, and their fetuses were collected 28, 32, 36, 39, and 42 days later. At each time point, genital ridges were dissected from the mesonephros and germ cells were isolated through magnetic-activated cell sorting using an anti-SSEA-1 antibody, and recovered germ cells were subjected to whole-genome bisulphite sequencing. Methylation levels were quantified using SeqMonk software by performing an unbiased analysis, and persistently methylated regions (PMRs) in each sex were determined to extract those regions showing 50% or more methylation. Most genomic elements underwent a dramatic loss of methylation from day 28 to day 36, when the lowest levels were shown. By day 42, there was evidence for the initiation of genomic re-methylation. We identified a total of 1456 and 1122 PMRs in male and female germ cells, respectively, and large numbers of transposable elements (SINEs, LINEs, and LTRs) were found to be located within these PMRs. Twenty-one percent of the introns located in these PMRs were found to be the first introns of a gene, suggesting their regulatory role in the expression of these genes. Interestingly, most of the identified PMRs were demethylated at the blastocyst stage.

**Conclusions:**

Our findings indicate that methylation reprogramming in pig germ cells follows the general dynamics shown in mice and human, unveiling genomic elements that behave differently between male and female germ cells.

## Background

Mammalian genomes undergo epigenetic reprogramming, which mostly involves the reprogramming of histone modifications and the erasure and re-establishment of DNA methylation [1]. In the mammalian life cycle, epigenetic reprogramming occurs at two time points: the first is during pre-implantation development, and the second, occurring in the germline, is when primordial germ cells (PGCs) migrate to the genital ridges [2, 3]. Some of the epigenetic marks established over these periods play a role in the activation and inactivation of certain genes, therefore having a potential impact on the transcriptome of an individual [4]. Although germline development has been the subject of intense research, most data so far have been provided by studies performed in mice, so inferences from available data must be made with caution. Recent studies have also examined DNA de-methylation during the reprogramming of germ cells in humans [5–8]. However, re-methylation in human germ cells has not been explored, and differences were detected with respect to mouse PGCs including their mitotic behaviour during the de-methylated period. The pig is a broadly used model for human, as pigs are evolutionarily closer to human than mice [9, 10] and both species share various physiologic and anatomic characteristics [11]. Some studies have identified key aspects of porcine germ cell methylation and development [12, 13], yet more extensive studies are necessary to fully understand the dynamics of epigenetic reprogramming. This information will help understand the mechanism of reprogramming of gonadal germ cells in mammals.

DNA methylation occurs across the whole genome and affects both regulatory gene expression elements such as promoters, gene bodies, exons and introns [14, 15], and transposable elements, such as short interspersed nuclear elements (SINEs), long interspersed nuclear elements (LINEs), and long terminal repeats (LTRs). In mice, germ cell reprogramming occurs between embryonic days E9 and E15, and by E13.5 germ cells are extremely hypomethylated, although a small amount of methylation persists [16, 17]. The global reprogramming that takes place at these stages is a complex process, triggered by the base excision repair mechanism among others [18], that results in the appropriate activation of the genes implicated in germline reprogramming, therefore enabling gametogenesis [19]. Some studies have shown that only intracisternal A particles (IAPs) as a repetitive sequence class remain substantially methylated across all stages of mouse germ cell reprogramming, while other transposable elements, such as LINE1s and SINEs, are largely reprogrammed [20–22]. Other authors have also identified resistant CGIs and non-CGI promoters that remain methylated in male and female E13.5 germ cells, and CGIs located close to an IAP showing high methylation levels across all developmental stages, suggesting that the genomic context or chromatin environment of IAPs can confer resistance against the erasure of neighbouring elements [22].

In humans, the general dynamics of germ cell methylation reprogramming are similar to those in mice but more prolonged in time. The reprogramming of human germ cells has been analysed between weeks 5 and 19 of gestation, with the lowest level of methylation detected between weeks 9 and 11 [6, 7]. Some of the evolutionary younger repetitive elements, such as SINE-Alu or LINE-L1PA, have been found to show high methylation levels at all stages of human germ cell development, and some regulatory regions (promoters, CGIs) have proven to be variably resistant to demethylation, thus suggesting their potential role in intergenerational epigenetic inheritance [7].

In the pig, migration of PGCs towards the genital ridges can be observed at the E18 stage of gestation, although they are only sexually dimorphic at E27, when the tunica albuginea can be histologically identified [23]. During this period, based on the analysis of specific sequences, including transposable elements and two imprinted loci, germ cells undergo methylation reprogramming until methylation resumes at E42 [13]. Previous studies have reported that, despite interspecies differences, DNA methylation changes are similar to those reported in mice but extended in time [12, 13], although global DNA re-methylation begins slightly earlier in porcine germ cells [12].

In the present study, we analysed methylation reprogramming in pig germ cells by whole-genome bisulphite sequencing and identify persistently methylated regions (PMRs) during the reprogramming of male and female germ cells, and we compared them with the PMRs found in gametes (sperm and oocytes) and blastocysts.

## Results

### Dynamic methylome analysis of porcine germ cells

**Fig. 1:**
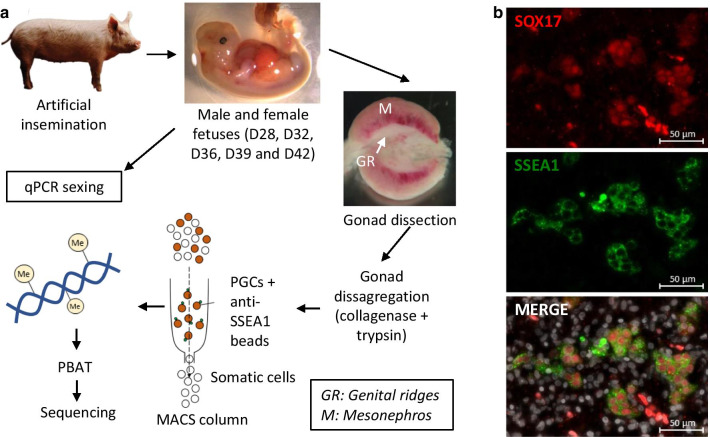
Experiment summary. **a** Porcine fetuses were collected on days D28, D32, D36, D39, and D42 after artificial insemination. Gonads were dissected and disaggregated, and genital ridges (GR) were extracted from the mesonephros (M). Germ cells were recovered with an anti-SSEA1 antibody on a MACS column and subjected to PBAT sequencing. **b** Immunodetection of SSEA1 and SOX17 in the genital ridges of porcine fetuses on Day 45 using anti-SSEA1 and anti-SOX17 antibodies. Nuclear staining using DAPI is shown in grey in the lower panel

Germ cells from male and female fetuses were isolated at different developmental stages (D28, D32, D36, D39, and D42) by MACS with the anti-SSEA1 antibody. Whole-genome bisulphite sequencing (WGBS) analysis of the isolated germ cells was performed using a post-bisulphite adaptor tagging (PBAT) technique [24] (Fig. 1a). First, we analysed whether SSEA1 was a good marker for the selection of porcine germ cells. SSEA1 expression has been reported in porcine germ cells at D22, D25, and D31 [12], but there were no clear data that SSEA1 was expressed on D42. Here, SSEA1 expression was evaluated by immunofluorescence on D45 gonads using the germ cell marker SOX17, showing a clear expression in the cytoplasm of all germ cells, and indicating that it is a good marker for germ cell purification in pig, at least over the developmental days included in this study (Fig. 1b).

**Fig. 2:**
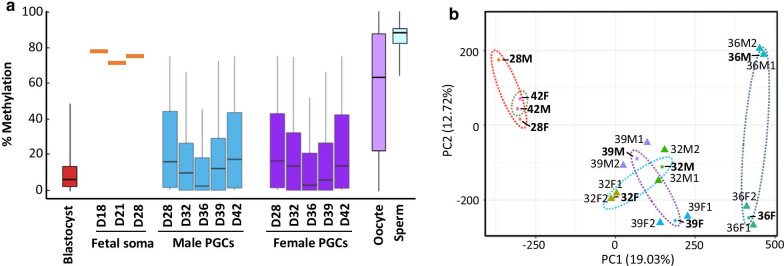
Global DNA methylation of porcine germ cells. **a** Boxplots representing the median DNA methylation levels (tiles of 100 CpG sites) detected in male (blue) and female (purple) germ cells at the different study time points (D28, D32, D36, D39, and D42). Boxes represent the interquartile range (Q1 to Q3), and whiskers show the minimum and maximum levels of methylation in each case. Methylation levels of pig blastocysts are from [26], mean methylation levels of fetal somatic tissue on D18, D21, and D28 reported by [25] are indicated in orange, and median methylation levels of oocytes and sperm are from [26]. **b** PCA of DNA methylation of tiles of 100 CpG sites of the samples included in this study

Post-bisulphite adaptor tagging (PBAT) libraries were made from germ cells recovered from pools of 3 pairs of genital ridges from three different fetuses per time point and sex. Two replicates per day and sex were used for whole-genome bisulphite sequencing, and samples showing inconsistencies were removed for further analysis, keeping 1 replicate for D28 and D42 for both male and female germ cells, and 2 replicates for D32, D36, and D39. The number of uniquely aligned sequences obtained from sequencing ranged from 7,048,430 to 46,675,415 and CpG methylation from 55 to 67% (Additional file 1). To obtain an unbiased measure of genome methylation, we fixed a minimum of 100 valid positions (CpGs) per window containing a minimum of 20 observations per feature, yielding a total of 327,583 tiles. We found that on day 28, overall DNA methylation, as evaluated from the median methylation of 100-CpG tiles, was 15.38% in male germ cells and 15.85% in female germ cells. Given that the methylation level in somatic cells of the pig fetus on day 28 has been reported at around 75% [25], it seems the main wave of DNA demethylation occurred in the germ cells before day 28 (Fig. 2a). We observed that in both male and female germ cells, median methylation levels of CpG sites declined after day 28, decreasing to 9.32% and 12.98%% on day 32 in male and female germ cells, respectively, and further falling to ~ 5.03% and 5.94% on day 36 with minimal difference between sexes (Fig. 2a). The lowest level of DNA methylation found in male and female germ cells at 36 days was the lowest DNA methylation level in the pig genome for any type of cell previously analysed. This hypomethylation was even lower than that reported in porcine blastocysts for transposons, transcripts, promoters, etc. [26]. This very hypomethylated genome resumes its re-methylation 3 days later in both male and female germ cells. On days 39 and 42, an increasing trend was noted in global methylation levels, revealing that re-methylation had already started, in accordance with previous studies performed by direct bisulphite sequencing in pig germ cells [13]. However, median methylation levels observed in sperm and oocytes are much higher (Fig. 2a) [26, 27], which indicates that the re-methylation process continues beyond day 42.

Overall methylation profiles were compared between days and sex by principal component analysis (PCA) using averaged methylation levels of CpG sites (Fig. 2b). The PC1 axis distributed samples according to developmental stages, whereas the PC2 axis generally separated male and female germ cells. There was a clear similarity between samples from the first and final time points (D28 and D42) of both sexes and also between samples from D32 and D39 germ cells. At the intermediate time point analysed (D36), we observed the greatest difference between male and female samples, which could be due to differences in the exact time point of the demethylation peak between sexes or may reflect differences in methylation related to sex determination/differentiation. This could also reflect slight differences in the developmental stages of the samples, due to the low number of replicates included in this study. Analysis of non-CpG methylation sites (CHC and CHH) revealed only a very discrete level of methylation ranging from 0.2 to 3.2% (data not shown), suggesting that pig gonad germ cells feature only marginal DNA methylation levels of non-CpG sites.

### Methylation reprogramming of functional genomic elements in porcine germ cells

**Fig. 3:**
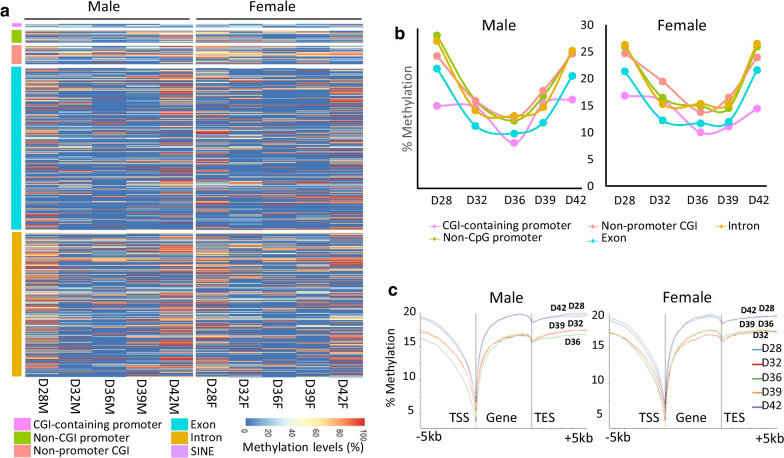
Methylome dynamics of functional genomic elements at the different time points for each sex. Number of replicates: D28M (*n* = 1), D32M (*n* = 2), D36M (*n* = 2), D39M (*n* = 2), D42M (*n* = 1), D28F (*n* = 1), D32F (*n* = 2), D36F (*n* = 2), D39F (*n* = 2), D42F (*n* = 1). **a** Heatmap showing levels of methylation of various genomic features. Each line corresponds to a single probe. High methylation levels are represented in red, and low methylation levels are shown in blue. **b** Line graph representing the mean level of methylation for the same genomic features represented in **a** on each day. **c** DNA methylation levels across gene bodies, including 5 kb upstream of the TSS and 5 kb downstream of the TES of all the genes examined in male and female germ cells

We further analysed the methylation dynamics of different functional genomic features. A general view of the methylation profile of these elements is shown in Fig. 3a, and their means is represented in Fig. 3b. A predominantly low level of methylation was observed in all the samples, with few tiles having a high methylation level (> 50%). Remarkably, the same clear pattern that we observed in the averaged methylation of CpG sites could be seen when looking at the methylation of functional genomic elements, for which a drop in the methylation levels from D28 to the lowest levels on D36, and then recovering it on D42 was observed. This general trend can be seen in all elements analysed in both sexes, although the demethylation trough was more pronounced in male germ cells. This could indicate differences in the start of re-methylation between male and female germ cells, again being cautions due to the low sample size of D28 and D42. In the case of CGI-containing promoters, the demethylation at D36 was even more marked: methylation fell to 8.54% in the case of males and 10.04% in females, whereas in promoters not located within a CGI the reduction of methylation was attenuated (Fig. 3b, Additional file 2). Introns and exons followed the same pattern of methylation reprogramming, with higher methylation levels detected in introns, as expected.

We next examined DNA methylation on and around the gene body region during de- and re-methylation. We found that the gene body follows a similar pattern in male and female germ cells (Fig. 3c), and that methylation was higher than that of adjacent regions. There was a noticeable hypomethylated region around the transcription start site (TSS), and a clear drop in methylation beyond the transcription end site (TES). As expected, gene bodies were more heavily methylated on D28 and D42 than D32, D36, and D39. Gene bodies still showed higher levels of methylation in comparison with the hypomethylation observed when we considered global methylation or CGIs. These levels were similar to those reported for the human germ cell gene body regions, which are also heavily demethylated at similar developmental stages [5, 6].

**Fig. 4:**
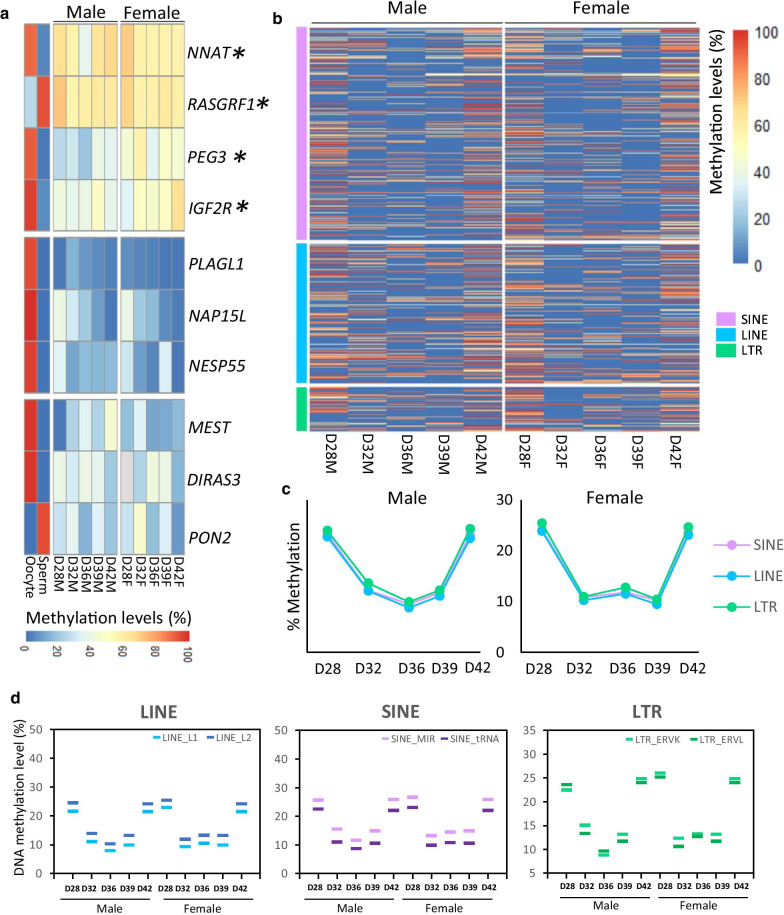
Methylome dynamics of functional genomic elements at the different time points for each sex. Number of replicates: D28M (*n* = 1), D32M (*n* = 2), D36M (*n* = 2), D39M (*n* = 2), D42M (*n* = 1), D28F (*n* = 1), D32F (*n* = 2), D36F (*n* = 2), D39F (*n* = 2), D42F (*n* = 1). **a** Clustered heatmap of imprinted genes in male and female germ cells on D28, D32, D36, D39, and D42. High methylation levels are represented in red, and low methylation levels are shown in blue. To the left, methylation levels in oocyte and sperm, data from [27]. **b** Heatmap showing the levels of methylation of the transposable elements analysed. Each line corresponds to a single probe. High methylation levels are represented in red, and low methylation levels are shown in blue. **c** Line graph representing the mean level of methylation for the same genomic features represented in **b** on each day. **d** Mean methylation levels of different subtypes of transposable elements: LINEs (left), SINEs (centre), and LTRs (right). The darker colour represents the evolutionary older element in each case

We also examined the methylation of imprinted and candidate-imprinted genes [27, 28]. The general assumption was that during germ cell development in the gonads, differentially methylated regions of imprinting genes would be thoroughly demethylated, as occurs in mice and humans [6], but we identified three clusters of imprinted genes in the pig depending on their methylation pattern: one of the strongly demethylated genes; one of the genes with variable dynamics; and another group of genes that retained methylation in the range 20 to 60% (Fig. 4a). Four of these genes seem to evade erasure of methylation (Fig. 4a, genes indicated with an asterisk), retaining methylation levels higher than 50%, including *IGF2R*, which is described to be demethylated in mice [29] but to resist demethylation in humans [7]. A general view of the methylation profiles of a demethylation-resistant gene (*RASGRF1*) is shown in Additional file 3.

We analysed separately the methylome dynamics of the X-chromosome, evaluating the methylation profiles of promoters containing CGIs of male and female germ cells. We observed that, in male germ cells, those promoters follow the general de- and re-methylation dynamics described above, whereas in female germ cells we detect higher methylation levels on D28, and we do not observe re-methylation after D36 (Additional file 4A, left panel). Most promoters showing around 20% of methylation at day 28 in female germ cells are hypomethylated from D36 onwards, suggesting that methylation of the inactive X-chromosome begins in later stages (Additional file 4A, right panel).

We also observed a different pattern of methylation between male and female germ cells in some genes involved in meiosis, by evaluating the averaged methylation levels of promoters of 70 genes involved in meiosis. In the case of male germ cells, we observed mean methylation levels around 22% in all days analysed. For female germ cells, we observed a different pattern: first, the mean methylation levels of the meiotic genes in germ cells are lower at all days analysed, and they drop from 15% at day 28 to ~ 9% from day 36 onwards (Additional file 4B, right panel). Moreover, we detected a set of genes that are highly methylated in males and demethylated in females, including *TRIP13* and *CDC25,* that belong to the ontology term of female meiosis I, *TRIP13* is required for sex body formation and synapsis of the sex chromosomes [30], and *CDC25* is required for resumption of oocyte meiosis [31]; *PIWIL1 and PIWIL2*, whose expression is enriched in human meiotic female germ cells [32]; *MEIKIN* is a key regulator of meiosis I kinetochore function, which is conserved from yeasts to humans [33]; *PPP2R1A* is essential for female meiosis and fertility in mice [34]; *PLK1* is essential during meiotic resumption in mice oocytes [35]; *DDX4* (*VASA*) is a marker of entry into meiosis of female mice germ cells [36]; *EHMT2* (*G9A*) is a mammalian H3K9 methyltransferase essential for an adequate meiotic prophase progression [37]; *PSMA8* in an associate proteasome essential for the degradation of meiotic proteins and the progression of meiosis I [38]; *CCNB2* participates in regulation of meiotic cell cycle in germ cells [39] and *NDC80*, which regulates meiotic spindle organization, chromosome alignment, and cell cycle progression in mouse oocytes [40] (Additional file 4B, left panel).

### Methylome dynamics of major transposable elements in porcine germ cells

Transposable elements comprise about half of the genome in mammals, and over 80% of pig protein-coding and lncRNA genes overlap with retrotransposon insertions [41]; thus, their regulation in terms of DNA methylation reprogramming is important to understand. We examined methylation patterns of the three major types of transposable elements: SINEs, LINEs, and LTRs. A summary of all elements analysed is provided in Table 1.

**Table 1:** Summary of the genomic elements analysed

General elements	Transposable elements		
	SINE	LINE	LTR
CpG-containing promoter	5s_Deu_L2	CR1	ERV1
Non-CpG promoter	MIR	L1	ERVK
Non-promoter CpG	tRNA	L2	ERVL
Exon		Penelope	ERVL_MaLR
Intron		RTE-BovB	
Imprinted genes		RTE-X	

DNA demethylation of the transposable elements analysed generally mirrored the global pattern (Fig. 4b), with mean levels of methylation of ~ 25% on D28 and D42 and dropping to ~ 10% and ~ 12% in male and female germ cells, respectively (Fig. 4c, Additional File 2). Interestingly, when we analysed the methylation patterns of subfamilies of retrotransposons during this wave of global demethylation, we observed that the evolutionarily younger and more active SINE and LTR transposable elements generally had higher levels of residual methylation than the evolutionarily older elements (Fig. 4d). However, this was not the case for the LINEs, for which the younger more active LINE1 showed lower levels of methylation than LINE2. We have also reported that methylation levels of LINE1 are lower than in LINE2 in pig and bovine blastocysts [26, 42].

### Demethylation-resistant genomic elements are candidate players in sex determination and early differentiation

**Fig. 5:**
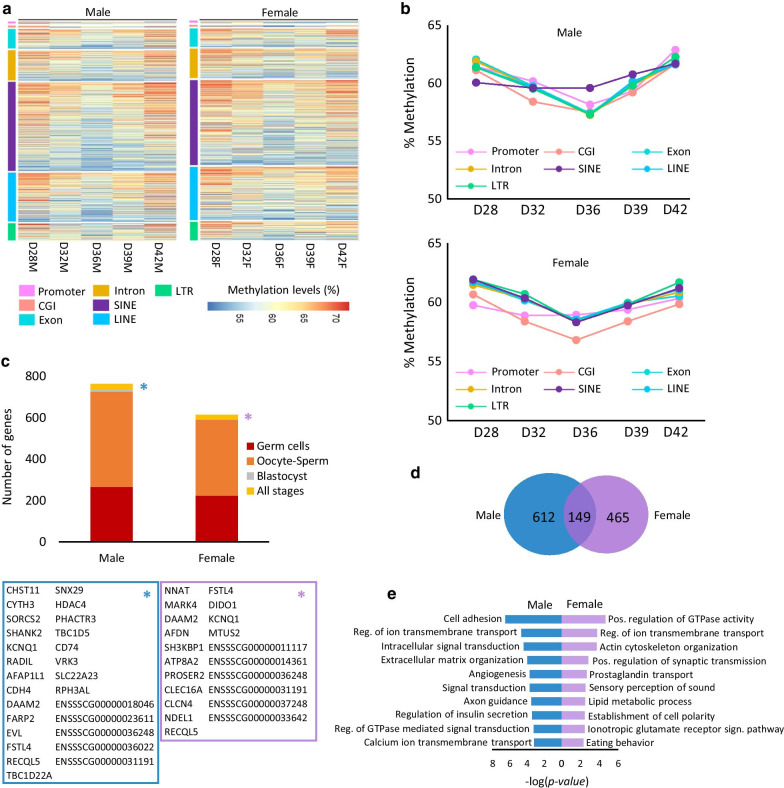
DNA methylation profile of the PMRs. Number of replicates: D28M (*n* = 1), D32M (*n* = 2), D36M (*n* = 2), D39M (*n* = 2), D42M (*n* = 1), D28F (*n* = 1), D32F (*n* = 2), D36F (*n* = 2), D39F (*n* = 2), D42F (*n* = 1). **a** Heatmap showing the levels of methylation of PMRs separated into their various genomic features. Each line corresponds to a single probe. **b** Line graphs representing the mean level of methylation for the same genomic features represented in **a** on each day. The upper panel corresponds to male PMRs and the lower panel to female PMRs. **c** Cumulative bar graph showing male and female genes located within the identified PMRs and their coincidence with PMRs in blastocyst [26] and oocyte and sperm [27]. Yellow section represents PMRs detected in all developmental stages. The lower panel shows the gene lists of genes located within PMRs throughout all developmental stages identified in male germ cells (left) and female germ cells (right). **d** Venn diagram showing genes containing PMRs in male and female PMRs. **e** Significantly enriched GO terms for persistently methylated genes in male (left panel, shown in blue) and female (right panel, shown in purple) germ cells

To identify regions resistant to demethylation, meaning their persistence in the globally demethylated genome, we assessed common 200 bp windows at all stages (D28, D32, D36, D39, and D42) for each sex, and selected those regions showing methylation of 50% or above, giving rise to 1,456 persistently methylated regions (PMRs) in male and 1,122 PMRs in female germ cells. We performed a similar analysis in pig blastocysts (data from [26]) and in oocyte and sperm (data from [27]) to identify common PMRs. The number of functional genomic elements included in these regions is shown in Table 2. A detailed report of all elements that remain persistently methylated in male germ cells and their overlap with PMRs in blastocyst and oocyte and/or sperm is provided in Additional file 5, and those persistently methylated in female germ cells and their corresponding overlap are included in Additional file 6. The summary of common PMRs is shown in Additional file 7. The global pattern of methylation of the elements included in the detected PMRs was similar in male and female germ cells (Fig. 5a). Nevertheless, mean methylation levels differed slightly between elements. Among PMRs in male germ cells, SINEs showed greater resistance to demethylation than other elements, which showed a slight decline in their methylation levels on D36 (Fig. 5b, upper panel). Regarding PMRs in female germ cells, we detected promoters as the elements that escaped demethylation completely but noted small variations in the remaining elements as well (Fig. 5b, lower panel). Remarkably, a sizeable proportion of the introns located within the detected PMRs were the first introns of a gene (20.63% and 20.92% in male and female germ cells, respectively), thus being candidates for regulating the expression of those genes, together with the great number of transposable elements that escape demethylation. Interestingly, a great proportion of those elements included in male and female PMRs are also highly methylated in oocyte and sperm, but only a small number of elements in blastocyst samples (Additional file 7). We identified a total of 21 and 27 genes in male and female germ cell PMRs, respectively, that remain methylated throughout all developmental stages (Fig. 5c), and some of them involved in functions related to intracellular protein transport, activation of GTPase activity, and nervous system development. Of note, histone deacetylase 4 (*HDAC4*), which plays a role in epigenetic repression, transcriptional regulation, and development events, was persistently methylated in male germ cells, blastocyst, and oocyte-sperm [43]. Four of the genes were detected in both male and female germ cells (*KCNQ1*, *DAAM2*, *FSTL4*, and *RECQL5*).

**Table 2:** Number of genomic elements detected in male and female PMRs

Element	Male	Female	Elements analysed
CGI-containing promoter	6	7	4180
Non-CGI promoter	102	66	20,043
Non-promoter CGI	76	51	20,915
Promoter-containing CGI	8	11	6024
Exon	936	696	365,797
Intron	1454	1128	359,350
Imprinted genes	2	3	10
Genes	763	614	24,232
SINE	4310	3250	1,044,910
LINE	2377	2037	684,309
LTR	839	710	204,271

To analyse the potential significance of our findings, we evaluated those genes located within all the identified PMRs. Most genes (80.2% and 75.3% in male and female germ cells, respectively) behaved as sex-specific (Fig. 5d), meaning these genes escape demethylation in one sex but not in the other. Accordingly, we detected 612 male-specific genes and 465 female-specific genes, a substantial proportion of those genes (67% in male germ cells and 74% in female germ cells) being overlapped by transposable elements identified as persistently methylated, with SINEs as the most prevalent features (Additional files 5 and 6). DAVID Gene Ontology analysis revealed enrichment in key functions, such as cell adhesion, signalling, and migration, regulation of insulin secretion and metabolic functions, signal transduction, ion transmembrane transport, actin cytoskeleton organization, eating behaviour, etc. (Fig. 5e). Also, Gorilla Ontology analysis was able to identify enrichment in RNA splicing, positive regulation of cell morphogenesis, and cellular response to a carbohydrate stimulus. We also identified 27 and 16 sex-specific genes in male and female PMRs that are also highly methylated in pig blastocysts (Additional files 5 and 6). Moreover, we found 156 and 143 genes containing PMRs in male and female germ cells, respectively, that are differentially expressed in male and female embryos during sex determination or express different isoforms in a sex-specific way in mice (Additional file 8) [44]. We also found that 45 of the genes containing PMRs identified in male germ cells and 48 in female germ cells are differentially expressed during sex determination in the cow (Additional file 8) [45]. Taken together, these observations support the idea that the genes here identified could play a role in sex determination and early differentiation, but another relevant functions are not discarded.

## Discussion

Epigenetic reprogramming of the germline is a crucial step in embryo development, as it is needed to re-establish the epigenetic status of the gametic genomes and thus reinitiate the developmental programme of the new individual [2]; it also presents a barrier in mammals to widespread epigenetic inheritance. Although a lot of research has been conducted in epigenetic reprogramming, information about this process in pig is still limited. The study of epigenetic events in the pig is important to unveil conserved mechanisms of germ cell reprogramming in mammals. In this systematic investigation of male and female porcine germ cells, we provide insight into the critical events that occur in this species during methylation reprogramming and identify those elements that escape demethylation.

We found that global DNA demethylation in pig germ cells is much more extensive than the first wave in preimplantation embryos, in agreement with reports of human germ cells [6]. Global DNA methylation in pig germ cells fell from D28 to D32, dramatically decreasing to 6% on day 36 in both male and female germ cells, with re-methylation started to be detected by D42. This indicates that re-methylation in female germ cells starts, at least partially, earlier than in mice. Although there is little information about the process of female germ cell re-methylation in pig, our results go in accordance with those from Hyldig et al.[13], where they describe a similar pattern of re-methylation in male and female porcine germ cells in a limited number of imprinted genes and transposable elements. Also, it has been described that re-methylation in human female germ cells starts at gestational week 11, even before than male germ cells. The 6% methylation level observed at D36 is the lowest reported in porcine cells and goes in accordance with the demethylation trough in humans [7]. Although the timing of reprogramming differs from the period required for DNA methylation reprogramming in mice [22] and humans [46], it takes place at analogous developmental stages, following the results of a similar study conducted in the porcine germline [13]. While the general profile of methylation reprogramming was similar in male and female germ cells, we observed a slightly different behaviour on D36. This difference could be related to variations in the exact time point of complete demethylation, as occurs in human germ cells, where female germ cells show their lowest methylation levels earlier than male germ cells [6]. Alternatively, it could reflect methylation differences related to sex determination/differentiation. Nevertheless, this information must be taken cautiously, as there could be slight differences in the developmental stages of the samples that would not be corrected due to the low number of replicates available. Therefore, further studies are needed to confirm these findings.

The global pattern of methylation here described was homogeneous across all genomic features analysed (CGIs, promoters, exons, introns, and transposable elements). Also, we noted that during global demethylation, methylation of the gene body and its adjacent regions also dropped significantly (Fig. 3c), similar to findings in humans [5, 6].

Despite the generally low levels of methylation, we were able to detect PMRs during germ cell reprogramming. This observation is important, as germline methylation reprogramming has been described as the main impediment to intergenerational epigenetic inheritance [47]. We identified genomic features located within those regions, which accordingly were able to avoid methylation erasure, as being potentially involved in this process, as described in mouse and human germ cells [6, 7, 22, 48]. To our knowledge, this is the first description of genome regions resistant to methylation erasure in the pig, and we believe it to be a good starting point for the investigation of the potential for epigenetic inheritance in livestock species. We identified 1,456 and 1,122 regions remaining persistently methylated (methylation at least 50%) in male and female germ cells, respectively, of which the majority were sex-specific. However, fewer than 3% of the PMRs also remain highly methylated in pig blastocysts, indicating that their methylation is erased during epigenetic reprogramming in the preimplantation embryo, thus making them unlikely mediators of intergenerational epigenetic inheritance.

Remarkably, a significant percentage of introns found to escape de-methylation were the first introns of a gene (~ 21%). Indeed, these elements have special functional roles, such as regulating the correct cytoplasmic localisation of some mRNAs [49], and inverse correlation has been described between DNA methylation of the first intron and gene expression across tissues [50]. Persistently methylated transposable elements (SINEs, LINEs, and LTRs) could also affect overlapping or neighbouring genes, with possible consequences on regulatory networks [51, 52]. We only identified 149 genes persistently methylated during the whole period of DNA methylation reprogramming in both male and female germ cells, being the vast majority of the genes located within PMRs (87.8%) sex-specific, remaining methylated only in one sex (612 in male and 465 in female germ cells). The majority of the identified genes showed overlap with highly methylated transposable elements, supporting the idea that transposable elements regulate their expression. Moreover, 27 and 16 of these sex-specific genes in male and female PMRs were also identified within blastocyst PMRs, and a substantial number of genes that are known to behave as sex-specific during sex determination in both cattle and mice, suggesting a possible epigenetic regulation of the expression of those genes [19, 30, 53].

However, some limitations of this study should be noted: due to the technical difficulties of obtaining germ cells, our sample size is very limited. The low number of samples (specially on D28 and D42) makes us take our results with caution, as it was not possible to correct the sample effect for those cases. Thus, we have not drawn a full map of the epigenetic reprogramming in the pig. Instead, we are establishing a preliminary base of the methylation dynamics during epigenetic reprogramming in the pig and the possible role of the genomic elements. Further large-scale studies would be needed to confirm the observations here described, and the role of the identified PMRs should be explored in higher depth.

## Conclusions

In conclusion, we found that methylation reprogramming in pig germ cells follows the general dynamics shown in mice and human and provides details of the methylome landscape differences between male and female porcine germ cells.

## Methods

### Sample collection

Animal experiments were performed following European legislation. Experiments were approved by the Committee on the Ethics of Animal Experiments of the INIA (permit number CEEA 2012/021). All chemicals were purchased from Sigma unless otherwise indicated.

Landrace-Large White sows were artificially inseminated with fresh semen and slaughtered at a commercial slaughterhouse 28 (*n* = 2), 32 (*n* = 2), 36 (*n* = 2), 39 (*n* = 2), and 42 (*n* = 2) days later. At each time point, uteri were collected and transported to the laboratory within an hour of slaughter. Fetuses were recovered from the uteri and dissected under a stereomicroscope in phosphate-buffered saline (PBS) free of calcium and magnesium, carefully detaching the mesonephros and genital ridges. Genital ridges were then washed in PBS and kept in fetal bovine serum (FBS) until the time of gonad dissociation. From each fetus, genomic DNA was extracted from the tail tip and sex determined by PCR using primers for the porcine amelogenin gene located on the sex chromosomes (forward primer CCAGCCAAACCTCCCTCTGCC, reverse primer CCCGCTTGGTCTTGTCTGTTGC) [45].

### Gonad dissection and germ cell isolation and immunofluorescence

Genital ridges were grouped by sex and developmental day (three pairs of genital ridges from three different fetuses per group), washed in PBS three times to clean them and remove blood, placed in a dish, and chopped up with a scalpel. The small pieces of gonads were transferred to collagenase IV (2 mg/ml in Dulbecco’s modified Eagle's medium (DMEM)) and incubated at 37ºC with periodic vortexing every 7 min. Next, the tissue fragments were washed with PBS and centrifuged for 5 min at 1100 revolutions per minute (rpm). The supernatant was removed and 1 ml of TrypLE Express (Gibco, Grand Island, NY, USA) was added to the pellet which was incubated for 3 min at 37ºC. TrypLE Express was then blocked with 3 ml of fetal calf serum (FCS) and the solution filtered through a cell strainer (40 µm) and centrifuged for 5 min at 1100 rpm. Germ cells were finally labelled with anti-SSEA1 (stage-specific embryonic antigen-1 antibody) microbeads (MiltenyiBiotec Inc., UK) and separated on MS columns in a mini Magnetic-activated cell sorting (MACS) separation unit (MiltenyiBiotec Inc., UK) following the manufacturer’s instructions.

For cryo-sections, fixed genital ridges were incubated in 30% sucrose in PBS overnight at 4ºC before mounting in the OCT compound. Cryo-sections were cut at 7 µm onto glass slides. Sections were left to air dry for 1–2 h before immunofluorescence. For immunofluorescence, slides were rinsed in PBS, followed by a 15-min permeabilization step with 1% Triton X100 in PBS. Next, sections were blocked for 60 min with blocking solution (5% BSA-10% Donkey serum in PBS). Primary antibodies were diluted in blocking solution [mouse anti-SSEA1 1:50 (Santa Cruz, sc‐21,702, USA) and goat anti-SOX17 1:500 (R&D systems, AF1924, Minneapolis, MN, USA)], and slides were incubated overnight at 4 °C. Secondary antibodies Alexa Fluor 555 donkey anti-mouse (Thermo, A-31570, USA) and Alexa Fluor 647 donkey anti-goat (Thermo, A-21447, USA), were used at concentrations of 1:500 and incubated with the slides for 1 h at RT. Finally, slides were mounted with Fluoroshield with DAPI (Sigma-Aldrich, F6057, MI, USA).

### Post-bisulphite adaptor tagging (PBAT) sequencing

Recovered germ cells were selected for whole-genome bisulphite sequencing using an adaptation of the post-bisulphite adapter tagging (PBAT) method [24], which does not discriminate between 5-methylcytosine (5mC) 5-hydroxymethylcytosine (5hmC). Two groups of germ cells from different fetuses per sex and stage were sequenced. Briefly, cells were collected in 10 µl of PBS and lysed in 15 µl lysis buffer containing 0.5% sodium dodecyl sulphate (SDS), 1 µl proteinase K, and EB, using the Imprint DNA modification kit (Sigma). The bisulphite converted DNA was eluted with elution buffer (EB) and one round of first-strand synthesis performed using a biotinylated oligo 1 (5-[Btn]CTACACGACGC-TCTTCCGATCTNNNNNNNNN-3). Samples were further treated with Exonuclease I, washed and eluted with EB, and subjected to solid-phase reversible immobilization (SPRI) purification and incubated with 20 µl Streptavidin Dynabeads to capture the biotinylated fraction of DNA. Second-strand synthesis was performed using oligo 2 (5′-TGCTGAACCGCTCTTCCGATCTNNNNNNNNN -3′), and samples were amplified for 15 PCR cycles using indexed iPCRTag reverse primers with KAPA HiFi HotStart DNA polymerase (Kapa Biosystems) and purified using SPRI beads (Agencourt Ampure XP bead). The quality of the libraries was assessed using high-sensitivity DNA chips in the Agilent bioanalyser, and the KAPA Library Quantification Kit for Illumina (KAPA Biosystems). Twenty libraries were generated for 100 bp single-end sequencing in a NextSeq500 sequencer (Illumina). A summary of the whole procedure is provided in Fig. 1a.

### Methylation analysis

For methylation analysis, we used *Sus scrofa* genome annotations (Sus scrofa v11.1.99, downloaded from Ensembl [54]). Transposable element annotations were downloaded from RepeatMasker [55], imprinted genes were taken from the Imprinted gene database [28] and from [27], and genes involved in meiosis were downloaded from UniProtKB [56]. Methylation levels in blastocyst were obtained from [26], and oocyte and sperm methylation levels were checked in data from [27]. Data quality was checked using FastQC software [57], and sample alignment was performed using Bismark software (v.0.19; Babraham Institute). Samples showing alignment rates lower than 50% and/or showing inconsistencies in their position-averaged methylation levels (high M-bias) were discarded from the analysis, keeping a total of 16 samples: D28M (*n* = 1), D32M (*n* = 2), D36M (*n* = 2), D39M (*n* = 2), D42M (*n* = 1), D28F (*n* = 1), D32F (*n* = 2), D36F (*n* = 2), D39F (*n* = 2), D42F (*n* = 1). A summary of the sequencing statistics of the analysed samples is included in Additional file 1. After filtering the samples, tiles were defined for each day and sex group (D28M, D28F, D32M, D32F, D36M, D36F, D39M, D39F, D42M, and D42F) in SeqMonk software (v1.45.4, Babraham Institute) using the Read Position Probe Generator Tool, setting a minimum of 1 read count per position, and 100 valid positions per window. Methylation was quantified as described by [42]. Briefly, a methylation quantitation pipeline was run over the resulting tiles, including those tiles with a minimum count of 1 per position, and at least 20 observations per feature, then combined using the mean. To remove tiles without data, we discarded tiles whose values were not between 0 and 100 in at least one data store, obtaining a total of 327,583 tiles. The methylation quantitation pipeline was run again over the new tiles, keeping features with a minimum count of 1 per position, and at least 20 observations per feature, and then normalized by matching the distribution of the transformed mean. To determine persistently methylated regions (PMRs) in all stages analysed for each sex, we followed the pipeline described in [5], evaluating common 200 bp windows containing at least 6 CpG sites in each stage (D28, D32, D36, D39, and D42), and selected those regions showing a methylation value of 50% or above in all samples, yielding a total of 1,456 PMRs in the male germ cells and 1,122 PMRs in the female germ cells.

## Supplementary information

**Additional file 1:** Summary of sequencing statistics of each sample.

**Additional file 2:** Mean methylation levels of genomic elements.

**Additional file 3:** General view of the imprinted gene *RASGRF1,* demethylation resistant in germ cells*.* Blue and red dots represent methylation reads. The lower panel shows the methylation levels detected in the differentially methylated region (DMR) in all samples analysed.

**Additional file 4:** (A) X-chromosome methylome dynamics. The left panel shows a heatmap representing levels of methylation of X chromosome promoters, each line corresponding to a single feature. High methylation levels are represented in red, and low methylation levels are shown in blue. To the right, line graph representing the mean level of methylation of the X-chromosome promoters on each sex and day. (B) Methylation dynamics of 70 meiosis-related genes. The left panel shows a heatmap representing levels of methylation of genes involved in meiosis, each line corresponding to a single feature. High methylation levels are represented in red, and low methylation levels are shown in blue. Clusters showing different patterns of methylation between male and female are zoomed in. To the right, line graph representing the mean level of methylation of the meiosis-related genes analysed on each sex and day.

**Additional file 5:** Genomic elements reported for male PMRs including their levels of methylation in each sample and their overlap with blastocyst and oocyte-sperm PMRs. The following tabs are included: (A) Promoters, (B) CGIs, (C) Exons, (D) Introns, (E) SINEs, (F) LINEs, (G) LTRs, and (H) Genes. Tab (D) includes information about intron position, tabs (E), (F), and (G) include the element subtype, and tab (H) includes the possible coincidence of a feature in female PMRs.

**Additional file 6:** Genomic elements included in female PMRs, including their levels of methylation in each sample and their overlap with blastocyst and oocyte-sperm PMRs. The following tabs are included: (A) Promoters, (B) CGIs, (C) Exons, (D) Introns, (E) SINEs, (F) LINEs, (G) LTRs, and (H) Genes. Tab (D) includes information about the position of the intron, tabs (E), (F), and (G) include the subtype of the element, and tab (H) includes the possible coincidence of a feature in male PMRs.

**Additional file 7:** Summary of the elements included in common PMRs between germ cells (male and female separately) and blastocyst (data from [26]) and sperm-oocyte (data from [27]).

**Additional file 8:** Summary of the genes identified as persistently methylated in male and female germ cells differentially expressed in mice and bovine embryos during sex determination or coding for different isoforms depending on the sex.

## Data Availability

All data generated or analysed during this study are included in this published article [and its additional information files]. Bisulphite-sequencing data have been deposited in the ArrayExpress database at EMBL-EBI (www.ebi.ac.uk/arrayexpress) under accession number E-MTAB-9326.
