# Supplementary material for: Genome-wide DNA methylation dynamics during epigenetic reprogramming in the porcine germline
Source: Clin Epigenetics. 2021 Feb 3;13:27. doi: 10.1186/s13148-021-01003-x (PMC7860200; doi:10.1186/s13148-021-01003-x)
Supplement: Supplementary file 1 — Additional file 1: Summary of sequencing statistics of each sample. [file 13148_2021_1003_MOESM1_ESM.docx]

| Sample | Number of germ cells (x10^4^) | Raw sequences | Uniquely aligned sequences | Mapping efficiency (%) | Duplicates | Analysed sequences | Total C's analysed | Methylation CpGs (%) |
| --- | --- | --- | --- | --- | --- | --- | --- | --- |
| D28M1 | 0.750 | 17124150 | 10934128 | 63.85 | 723539 | 10210589 | 198945268 | 54.8 |
| D28F1 | 1.225 | 23517493 | 15743424 | 66.94 | 1445565 | 14297859 | 269982604 | 62.9 |
| D32M1 | 1.160 | 72387016 | 46675415 | 64.48 | 7807031 | 38868384 | 753172668 | 61.9 |
| D32M2 | 1.250 | 15164392 | 9728923 | 64.16 | 407010 | 9321913 | 182665931 | 63.4 |
| D32F1 | 1.250 | 24636689 | 15577820 | 63.23 | 1180797 | 14397023 | 276487970 | 53.8 |
| D32F2 | 1.275 | 17142538 | 11054326 | 64.48 | 464238 | 10590088 | 208421058 | 54.5 |
| D36M1 | 1.250 | 20575648 | 13425005 | 65.25 | 1057987 | 12367018 | 239783877 | 67 |
| D36M2 | 1.200 | 11078071 | 7048430 | 63.63 | 267062 | 6781368 | 134313746 | 66.2 |
| D36F1 | 2.500 | 15663899 | 10058370 | 64.21 | 573027 | 9485343 | 183381608 | 66.2 |
| D36F2 | 2.400 | 14984486 | 9474357 | 63.23 | 382476 | 9091881 | 178153266 | 63.7 |
| D39M1 | 2.700 | 36410837 | 21919696 | 60.20 | 2001405 | 19918291 | 376629945 | 62.9 |
| D39M2 | 2.500 | 15953340 | 9925664 | 62.22 | 438162 | 9487502 | 184716072 | 60.6 |
| D39F1 | 0.750 | 18648727 | 11184665 | 59.98 | 490500 | 10694165 | 205853765 | 60 |
| D39F2 | 0.850 | 12772614 | 8044329 | 62.98 | 295941 | 7748388 | 155158977 | 54.1 |
| D42M2 | 2.000 | 17330635 | 10939953 | 63.12 | 491921 | 10448032 | 211239506 | 60.8 |
| D42F2 | 1.150 | 15378534 | 9867318 | 64.16 | 525163 | 9342155 | 188760142 | 65.5 |

**Additional File 1**. Sample alignment report
