# Supplementary material for: Genome-wide DNA methylation dynamics during epigenetic reprogramming in the porcine germline
Source: Clin Epigenetics. 2021 Feb 3;13:27. doi: 10.1186/s13148-021-01003-x (PMC7860200; doi:10.1186/s13148-021-01003-x)
Supplement: Supplementary file 2 — Additional file 2: Mean methylation levels of genomic elements. [file 13148_2021_1003_MOESM2_ESM.docx]

|  | Male | | | | |  | Female | | | | |
| --- | --- | --- | --- | --- | --- | --- | --- | --- | --- | --- | --- |
| Element | D28 | D32 | D36 | D39 | D42 |  | D28 | D32 | D36 | D39 | D42 |
| CGI-containing promoter | 15.51 | 15.25 | 8.54 | 15.96 | 16.69 |  | 16.87 | 16.03 | 10.04 | 11.08 | 14.46 |
| Non-CGI promoter | 28.74 | 16.49 | 12.7 | 17.31 | 25.69 |  | 26.04 | 16.48 | 14.94 | 14.55 | 26.01 |
| Non-promoter CGI | 24.91 | 16.49 | 13.31 | 18.36 | 25.32 |  | 24.75 | 19.52 | 13.81 | 16.56 | 24.01 |
| Exon | 22.52 | 11.73 | 10.31 | 12.36 | 21.14 |  | 21.4 | 12.26 | 11.72 | 11.99 | 21.64 |
| Intron | 27.69 | 14.66 | 13.63 | 15.27 | 25.87 |  | 26.37 | 15.31 | 15.37 | 15.38 | 26.56 |
| SINE | 23.37 | 12.24 | 9.49 | 11.76 | 23.09 |  | 24.07 | 10.83 | 11.79 | 10.31 | 23.53 |
| LINE | 22.73 | 12.12 | 8.8 | 11.08 | 22.44 |  | 23.84 | 10.26 | 11.51 | 9.47 | 23.06 |
| LTR | 23.95 | 13.6 | 9.91 | 12.22 | 24.31 |  | 25.43 | 10.94 | 12.76 | 10.42 | 24.62 |

**Additional File 2**. Mean methylation levels of genomic elements.
