# Supplementary figures and images for: Genome-wide DNA methylation dynamics during epigenetic reprogramming in the porcine germline
Source: Clin Epigenetics. 2021 Feb 3;13:27. doi: 10.1186/s13148-021-01003-x (PMC7860200; doi:10.1186/s13148-021-01003-x)

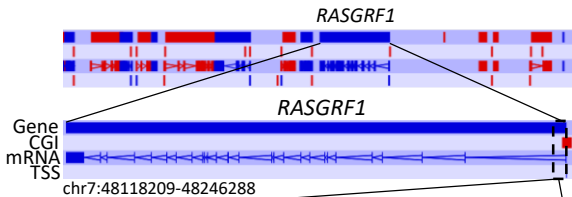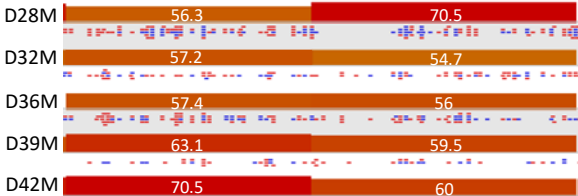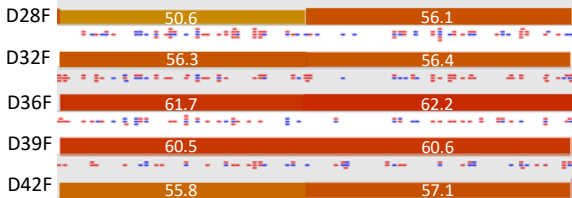

Supplement: Supplementary file 3 — Additional file 3: General view of the imprinted gene RASGRF1, demethylation resistant in germ cells. Blue and red dots represent methylation reads. The lower panel shows the methylation levels detected in the differentially methylated region (DMR) in all samples analysed. [file 13148_2021_1003_MOESM3_ESM.pdf]

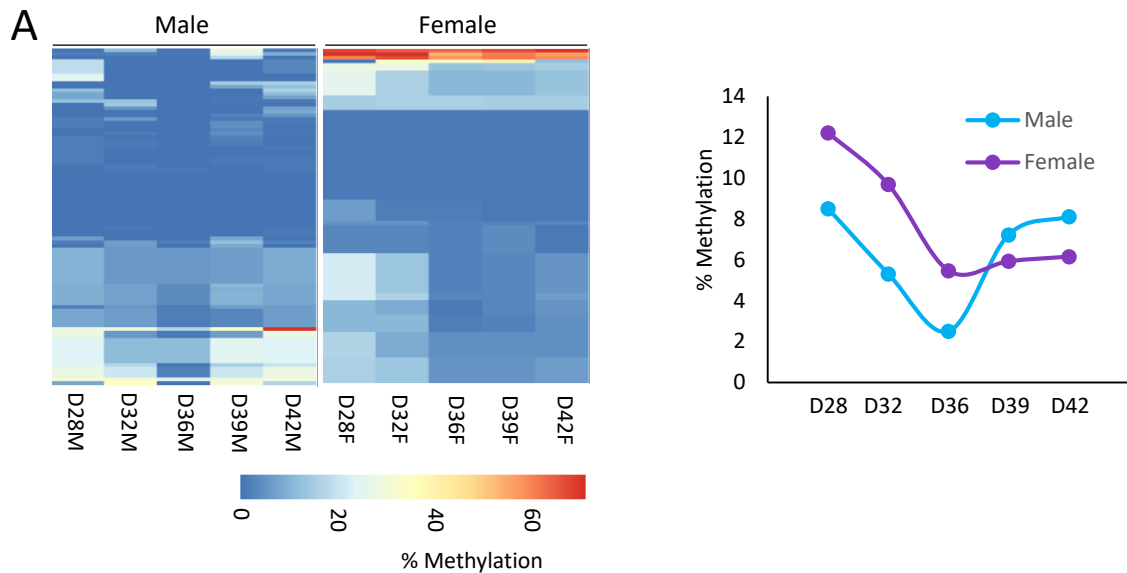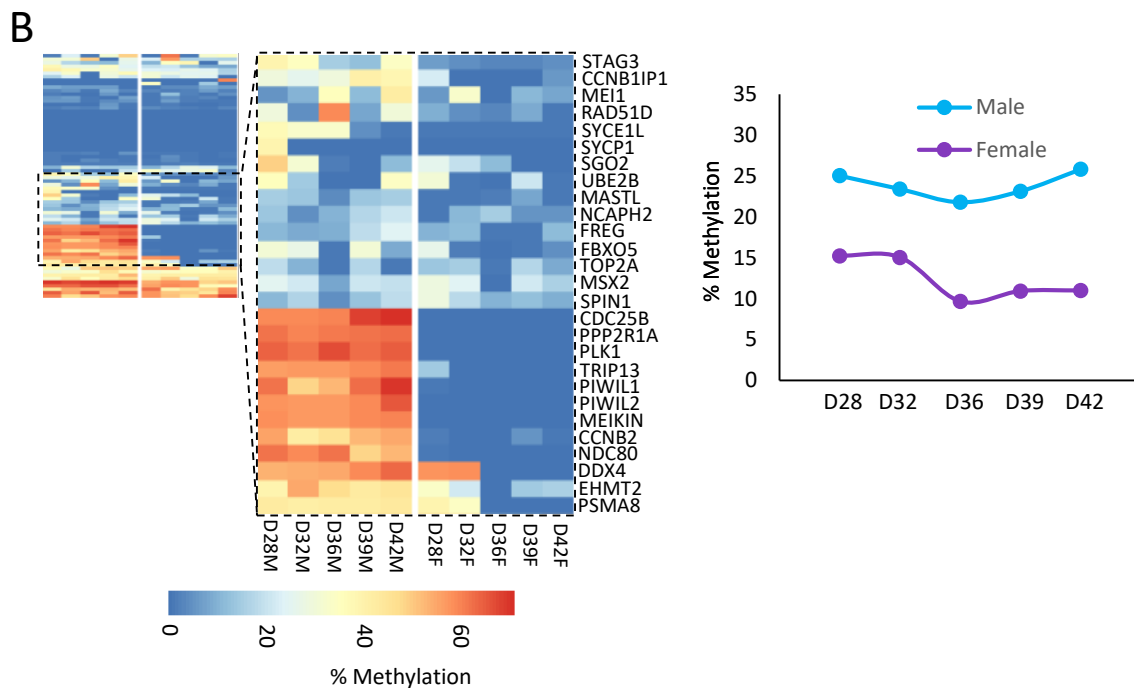

Supplement: Supplementary file 4 — Additional file 4: (A) X-chromosome methylome dynamics. The left panel shows a heatmap representing levels of methylation of X chromosome promoters, each line corresponding to a single feature. High methylation levels are represented in red, and low methylation levels are shown in blue. To the right, line graph representing the mean level of methylation of the X-chromosome promoters on each sex and day. (B) Methylation dynamics of 70 meiosis-related genes. The left panel shows a heatmap representing levels of methylation of genes involved in meiosis, each line corresponding to a single feature. High methylation levels are represented in red, and low methylation levels are shown in blue. Clusters showing different patterns of methylation between male and female are zoomed in. To the right, line graph representing the mean level of methylation of the meiosis-related genes analysed on each sex and day. [file 13148_2021_1003_MOESM4_ESM.pdf]
