# Supplementary material for: Genome-wide DNA methylation dynamics during epigenetic reprogramming in the porcine germline
Source: Clin Epigenetics. 2021 Feb 3;13:27. doi: 10.1186/s13148-021-01003-x (PMC7860200; doi:10.1186/s13148-021-01003-x)
Supplement: Supplementary file 7 — Additional file 7: Summary of the elements included in common PMRs between germ cells (male and female separately) and blastocyst (data from [26]) and sperm-oocyte (data from [27]). [file 13148_2021_1003_MOESM7_ESM.docx]

**Additional File 7**. Summary of elements included in common PMRs between PGCs and blastocyst and oocyte-sperm.

|  | **Male PGCs** | | **Female PGCs** | |
| --- | --- | --- | --- | --- |
| Element | Blastocysts (common) | Sperm + Oocyte (common) | Blastocysts (common) | Sperm + Oocyte (common) |
| CGI-containing promoter | 0 | 3 | 0 | 5 |
| Non-CGI promoter | 0 | 56 | 1 | 38 |
| Non-promoter CGI | 4 | 43 | 0 | 19 |
| Promoter-containing CGI | 1 | 5 | 2 | 6 |
| Exon | 0 | 651 | 0 | 479 |
| Intron | 12 | 1022 | 17 | 738 |
| Imprinted genes | 0 | 0 | 1 | 2 |
| SINE | 1 | 2734 | 0 | 2057 |
| LINE | 0 | 1648 | 0 | 1397 |
| LTR | 0 | 544 | 0 | 443 |
| Genes | 37 | 477 | 23 | 405 |
| - Sex specific genes | 27 | 373 | 16 | 311 |
| - PMRs in both sexes | 10 | 104 | 7 | 94 |
